# Neural mechanisms of language development in infancy

**DOI:** 10.1111/infa.12540

**Published:** 2023-03-21

**Authors:** Scott Huberty, Christian O’Reilly, Virginia Carter Leno, Mandy Steiman, Sara Webb, Mayada Elsabbagh, The BASIS Team

**Affiliations:** ^1^ Montreal Neurological Institute McGill University Montreal Quebec Canada; ^2^ AI Institute University of South Carolina Columbia South Carolina USA; ^3^ Institute of Psychiatry, Psychology and Neuroscience King's College London London UK; ^4^ Center on Child Health, Behavior and Development Seattle Children's Research Institute Seattle Washington USA; ^5^ Centre for Brain and Cognitive Development London UK

## Abstract

Understanding the neural processes underpinning individual differences in early language development is of increasing interest, as it is known to vary in typical development and to be quite heterogeneous in neurodevelopmental conditions. However, few studies to date have tested whether early brain measures are indicative of the developmental trajectory of language, as opposed to language outcomes at specific ages. We combined recordings from two longitudinal studies, including typically developing infants without a family history of autism, and infants with increased likelihood of developing autism (infant‐siblings) (*N* = 191). Electroencephalograms (EEG) were recorded at 6 months, and behavioral assessments at 6, 12, 18, 24 and 36 months of age. Using a growth curve model, we tested whether absolute EEG spectral power at 6 months was associated with concurrent language abilities, and developmental change in language between 6 and 36 months. We found evidence of an association between 6‐month alpha‐band power and concurrent, but not developmental change in, expressive language ability in both infant‐siblings and control infants. The observed association between 6‐month alpha‐band power and 6‐month expressive language was not moderated by group status, suggesting some continuity in neural mechanisms.

## 1 INTRODUCTION

The course of early language development is known to vary between individuals (Brignell et al., 2017, 2018), particularly so in neurodevelopmental conditions (Fair et al., 2012; Jeste & Geschwind, 2014; Pickles et al., 2014). In autism spectrum disorder (ASD) for example, some children develop typical language, some remain minimally verbal (Tager‐Flusberg & Kasari, 2013), and for others, language development will follow a trajectory that is somewhere in between. For example, while language regression appears to be common in autism, many of these children will still exhibit typical language ability by childhood (Lord et al., 2004; Pickles et al., 2009). Still, developing functional language in infancy is associated with overall better language and communication abilities later in life (Baghdadli et al., 2018; Mawhood et al., 2000; Pickles et al., 2022; Szatmari et al., 2016), highlighting the need to support children in meeting early developmental milestones.

Language delays appears to be at least somewhat heritable, and it tends to be more common in children with a family history of autism, suggesting an association with ASD risk as opposed to solely with autistic diagnosis (Bishop et al., 1995; Stromswold, 2001). For example, infants born into families with an older autistic child (infant‐siblings) are more likely to experience early language delays, even if they themselves do not develop autism (Elsabbagh, 2020; Gamliel et al., 2009; Hudry et al., 2014; Longard et al., 2017; Marrus et al., 2018; Messinger et al., 2013; Ozonoff et al., 2011; Swanson et al., 2017; Szatmari, et al., 2016). These delays may extend beyond infancy; school‐age children who were born into families with a history of autism are also more likely to exhibit speech and language difficulties (Miller et al., 2016).

Given the heterogeneity of language development, particularly as it relates to neurodevelopmental conditions such as autism, an increasing number of studies have aimed to understand its underlying functional brain networks. Measures of local neuronal synchrony, such as EEG spectral power, reflect the activation of organized neuronal assemblies, and changes in EEG power across infancy are thought to reflect cortical maturation of the developing brain. EEG power in functionally distinct frequency ranges has been linked to the development of various cognitive processes, including attention (Klimesch, 2012), memory (Herrmann et al., 2004), motor control (Saby & Marshall, 2012), and language (Benítez‐Burraco & Murphy, 2016). While there has been some discrepancy across studies in language‐related neural markers, EEG power in the alpha, beta, and gamma frequency ranges has been observed to be associated with expressive language in both typical development (Brito et al., 2016; Cantiani et al., 2019; Pierce et al., 2021), and in neurodevelopmental and genetic conditions (Benasich et al., 2008; Jones et al., 2020; Levin et al., 2017; Wilkinson et al., 2019; Wilkinson et al., 2019). Neural oscillations in these frequency bands are thought to facilitate verbal fluency and comprehension (Giraud & Poeppel, 2012; Rojas & Wilson, 2014, 2014van Driel et al., 2014; Wojtecki et al., 2017), and may help to explain the variation in language ability in autism and associated conditions (Benítez‐Burraco & Murphy, 2016).

Still, prior infant studies have only tested whether measures of early cortical activity such as EEG power were related to language at a single time during development. Therefore, our knowledge of how these measures relate to ensuing language development remains limited. Variation in developmental trajectories needs to be considered when accounting for outcome heterogeneity in developmental conditions (Elsabbagh, 2020; Karmiloff smith 1998), and better knowledge of the predictors of early language development may help to promote better outcomes.

To address this knowledge gap, we combined data from two longitudinal infant‐sibling cohorts to assess whether spectral power at 6 months is associated with concurrent language, and/or the trajectory of language development over the first 3 years of life. In a previous study, we observed lower EEG power in the canonical frequency bands in infant‐siblings (Huberty et al., 2021). Building upon these findings, we now explore the continuity in the underlying mechanisms linking EEG power with language development and whether early differences in brain networks may affect later language development.

Based on previous studies that reported associations between language and spectral power in the theta (Jones et al., 2020; Pierce et al., 2021; Wilkinson et al., 2019), alpha (Levin et al., 2017; Wilkinson et al., 2019), and gamma (Benasich et al., 2008; Cantiani et al., 2019; Wilkinson et al., 2019) bands, we hypothesized that EEG power in these bands would be positively associated with concurrent and developmental expressive but not receptive language ability. While one previous study found that EEG power in the beta band was associated with language in infant‐siblings but not controls (Wilkinson et al., 2019), it is possible that the lack of observed association in the control infants was due to reduced variation in language outcomes, leading to reduced power to detect effects. As such, we did not hypothesize that associations between EEG power in any frequency bands and language would be moderated by group status. We accounted for biological sex and non‐verbal cognitive ability throughout, as these factors are known to influence both spectral power and language development (Bedford et al., 2016; Zambrana et al., 2012).

## 2 METHODS

### 2.1 Participants

We used data from EEG‐IP (the International Infant EEG Data Integration Platform; van Noordt et al., 2020) which contains 410 EEG recordings from 191 infants from two longitudinal infant‐sibling studies (Birkbeck, University of London: 188; University of Washington in Seattle: 222), including repeated measurements of EEG (6–18 months) and behavioral assessments (6–36 months). Both studies were conducted according to guidelines laid down in the Declaration of Helsinki, with written informed consent obtained from a parent or guardian for each child before any assessment or data collection. All procedures involving human subjects in this study were approved by Institutional Review boards at the respective institution (Birkbeck University and The University of Washington). See supplementary Table S1 for additional information on the number of Mullen assessments across visits.

Participants were either considered to have increased likelihood of developing autism by virtue of having an older sibling with a clinical diagnosis of autism (infant‐siblings), or at typical‐likelihood given the absence of a family history of autism (hereby referred to as typically developing control infants). The autism diagnosis of proband siblings was confirmed in both studies (see Jones et al., 2016; Orekhova et al., 2014). Out of the 191 participants, this analysis includes the 131 participants with both a 6‐month EEG recording and available Mullen Scales of Early Learning (MSEL) assessments; 68 typically developing controls (33 females, 35 males), and 63 infant‐siblings (29 females, and 34 males). 21 of the participants received a diagnosis of ASD (12 males, 9 females)^1^ based on 24‐ or 36‐month ADOS assessment and clinical judgment (Jones et al., 2016; Orekhova et al., 2014). 13 participants could not be included because their EEG recordings did not include the resting state paradigm, and 5 further participants could not be included due to file issues with their EEG recording. Finally, 5 participants could not be included because they were not administered the MSEL assessment. Remaining participants provided at least 32 s of clean resting‐state EEG collected at 6 months (*N* = 37/191 excluded; Salinsky et al., 1991; Gasser et al., 1985), and received the MSEL assessment at a minimum of one of the following time points: 6, 12, 18, 24, and 36 months. To assure that excluded participants did not differ on demographic factors such as group or autism outcome, 2 chi‐square tests of independence were performed to examine the relationship between exclusion status, and group (infant‐sibling, control) and ASD‐outcome respectively. Both tests were non‐significant (asd‐outcome: *X* ^2^ (1, *N* = 191) = 0.99, *p* = 0.32; group: *X* ^2^ (1, *N* = 191) = 0.64, *p* = 0.42). Table 1 presents a summary of the participants that were included in this analysis.

**TABLE 1:** Sample demographics; sample size (*N*), descriptive statistics for age at visit, MSEL Expressive Language Age‐Equivalent Scores (AE), MSEL Receptive Language AE, and MSEL nonverbal AE.

	Whole sample		Infant‐siblings	Control infants	*t*‐test (infant‐siblings v controls)
	N (London/Seattle)	Mean, SD, range	Mean, SD, range	Mean, SD, range	
6‐month visit	(55/76)		*n* = 63	*n* = 68	
Age in months at assessment		6.6, (1.01; 6–10)	6.62, (1.04; 6–10)	6.57, (1; 6–10)	*T* (129) = 0.256 (*p* = 0.798)
MSEL expressive language AE		6.05, (1.82; 3–14)	5.76, (1.75; 3–14)	6.32, (1.86; 3–14)	*T* (129) = −1.775 (*p* = 0.078)
MSEL receptive language AE		5.99, (1.77; 1–14)	5.65, (2.05; 1–14)	6.31, (1.4; 3–11)	***T* (129) = –2.162 (*p* = 0.032)**
MSEL nonverbal AE		7.39, (1.88; 4.5–16)	7.15, (1.89; 4.5–16)	7.6, (1.86; 4.5–13)	*T* (129) = −1.378 (*p* = 0.170)
12‐month visit	(54/72)		*n* = 63	*n* = 63	
Age in months at assessment		12.79, (1.35; 11–18)	12.83, (1.43; 11–18)	12.75, (1.28; 11–16)	*T* (124) = 0.328 (*p* = 0.744)
MSEL expressive language AE		12.28, (2.95; 7–22)	11.68, (3.1; 7–20)	12.87, (2.68; 7–22)	***T* (124) = –2.306 (*p* = 0.023)**
MSEL receptive language AE		12.33, (3.06; 5–24)	12.06, (3.35; 5–24)	12.6, (2.74; 5–22)	*T* (124) = −0.990 (*p* = 0.324)
MSEL nonverbal AE		15.42, (1.98; 8.5–23)	15.02, (2.05; 8.5–23)	15.83, (1.84; 12–20)	***T* (123) = –2.336 (*p* = 0.021)**
18‐month visit	(0/70)		*n* = 33	*n* = 37	
Age in months at assessment		18.1, (0.54; 17–20)	18.09, (0.46; 17–20)	18.11, (0.61; 17–20)	*T* (68) = −0.131 (*p* = 0.896)
MSEL expressive language AE		17.33, (3.9; 5–26)	17.91, (4.33; 8–26)	16.81, (3.45; 5–21)	*T* (68) = 1.179 (*p* = 0.242)
MSEL receptive language AE		18.97, (4.62; 10–28)	18.33, (4.73; 10–28)	19.54, (4.5; 10–27)	*T* (68) = −1.093 (*p* = 0.278)
MSEL nonverbal AE		19.66, (1.83; 15.5–24.5)	19.44, (1.9; 15.5–24)	19.85, (1.77; 16–24.5)	*T* (68) = −0.93 (*p* = 0.351)
24‐month visit	(53/36)		*n* = *60*	*n* = 29	
Age in months at assessment		23.99, (0.87; 21–27)	24.05, (0.98; 21–27)	23.86, (0.58; 23–25)	*T* (87) = 0.952 (*p* = 0.344)
MSEL expressive language AE		24.5, (5.94; 10–36)	23.47, (6.05; 10–35)	26.71, (5.11; 16–36)	***T* (86) = –2.459 (*p* = 0.016)**
MSEL receptive language AE		26.26, (6.28; 10–46)	24.88, (6.15; 10–34)	29.33, (5.53; 13–46)	***T* (85) = –3.217 (*p* = 0.002)**
MSEL nonverbal AE		25.61, (3.26; 17.5–32.5)	25.15, (3.21; 17.5–32)	26.65, (3.2; 21–32.5)	***T* (85) = –2.01** **(*p* = 0.047)**
36‐month visit	(54/0)		*n* = 29	*n* = 25	
Age in months at assessment		37.24, (2.7; 32–53)	37.28, (3.51; 32–53)	37.2, (1.29; 35–41)	*T* (52) = 0.102 (*p* = 0.919)
MSEL expressive language AE		40.83, (9.07; 9–60)	37.34, (9.09; 9–55)	44.88, (7.3; 28–60)	***T* (52) = –3.321 (*p* = 0.002)**
MSEL receptive language AE		39.37, (8.23; 8–57)	36.76, (8.32; 8–53)	42.4, (7.15; 27–57)	***T* (52) = –2.649 (*p* = 0.011)**
MSEL nonverbal AE		40.36, (7.15; 21–59.5)	38.73, (7.99; 21–59.5)	42.18, (5.69; 27.5–55.5)	*T* (51) = −1.78 (*p* = 0.079)

*Note*: Bold text in the *t*‐test column indicates statistical significance at *p* < 0.05.

### 2.2 Measures

#### 2.2.1 MSEL (Mullen, Eileen, n.d.; 1995)

The MSEL is a standardized developmental measure that can be used from birth to 68 months. Raw, standard (T‐scores), age‐equivalent (AE) scores, and percentile scores can be generated for Gross Motor, Fine Motor, Visual Reception, Receptive Language, and Expressive Language scales. It directly assesses the child, but allows for parent report for certain items if the child does not exhibit the targeted behavior during the assessment period. For children with autism or developmental delays, AE scores of expressive and receptive language ability are sometimes preferable to avoid the floor effect that standard scores exhibit (Akshoomoff, 2006; Munson et al., 2008). To examine specificity of effects to language, a nonverbal age‐equivalent score (nonverbal AE) was calculated for each participant by averaging the fine motor and visual reception AE scores at each time point. In the London sample, participants were administered the MSEL at 6, 12, 24, and 36 months. In the Seattle sample, participants were administered the MSEL at 6, 12, 18, and 24 months, with the exception that most of the control infants did not receive the MSEL at 24 months.

#### 2.2.2 EEG collection

EEG was collected using Electrical Geodesics NetStation software and 128‐channel Hydrocel nets (Electrical Geodesics Inc., Eugene, OR). Resting‐state EEG (rs‐EEG) was collected while infants sat on their caregiver's lap and were presented videos on a monitor in a dark room. The two studies used similar videos, consisting of a set of age‐appropriate, brightly‐colored toys moving and producing sounds and a set with a woman facing the camera and singing nursery rhymes. The London study also used a third set of videos showing age‐appropriate toys being activated by a hand. The two video sets in the Seattle sample lasted 60 s each while the three video‐sets on the London sample lasted 30–40 s each.

#### 2.2.3 Data standardization and reduction

Data was pre‐processed using the EEG‐IP Lossless Pipeline (EEG‐IP‐L; https://github.com/BUCANL/EEG‐IP‐L; Desjardins et al., 2020), which includes systematic pre‐processing procedures for identifying and annotating unreliable EEG signals.

The EEG‐IP‐L pipeline harmonizes data recordings by implementing standardized data quality control. The pipeline first addresses differences across datasets by interpolating the EEG to a common montage, re‐referencing to the average, and filtering with a 1 Hz high‐pass and a notch filter (London: 49–51 Hz, Seattle: 59–61 Hz). Then, it flags time periods and channels with outlying variance. The rest of the quality assessment uses confidence intervals of signal properties within each recording to flag both time periods and channels with extreme variations. Each time new channels are flagged, the EEG is re‐referenced using an average interpolated reference excluding every channel flagged so far. Following the scalp channel assessment, an Adaptive Mixture Independent Component Analysis (AMICA; Palmer et al., 2011) is performed and components associated with environmental or physiological noise are removed. The application of this pipeline on EEG‐IP resulted in an average channel retention ranging between 77% and 82%, and produced similar power spectrum densities and data rejection rates across sites (van Noordt et al., 2020). For more detailed information on the processing and standardization of data in EEG‐IP, please refer to our previously published manuscript (van Noordt et al., 2020).

#### 2.2.4 EEG power estimation

**FIGURE 1:**
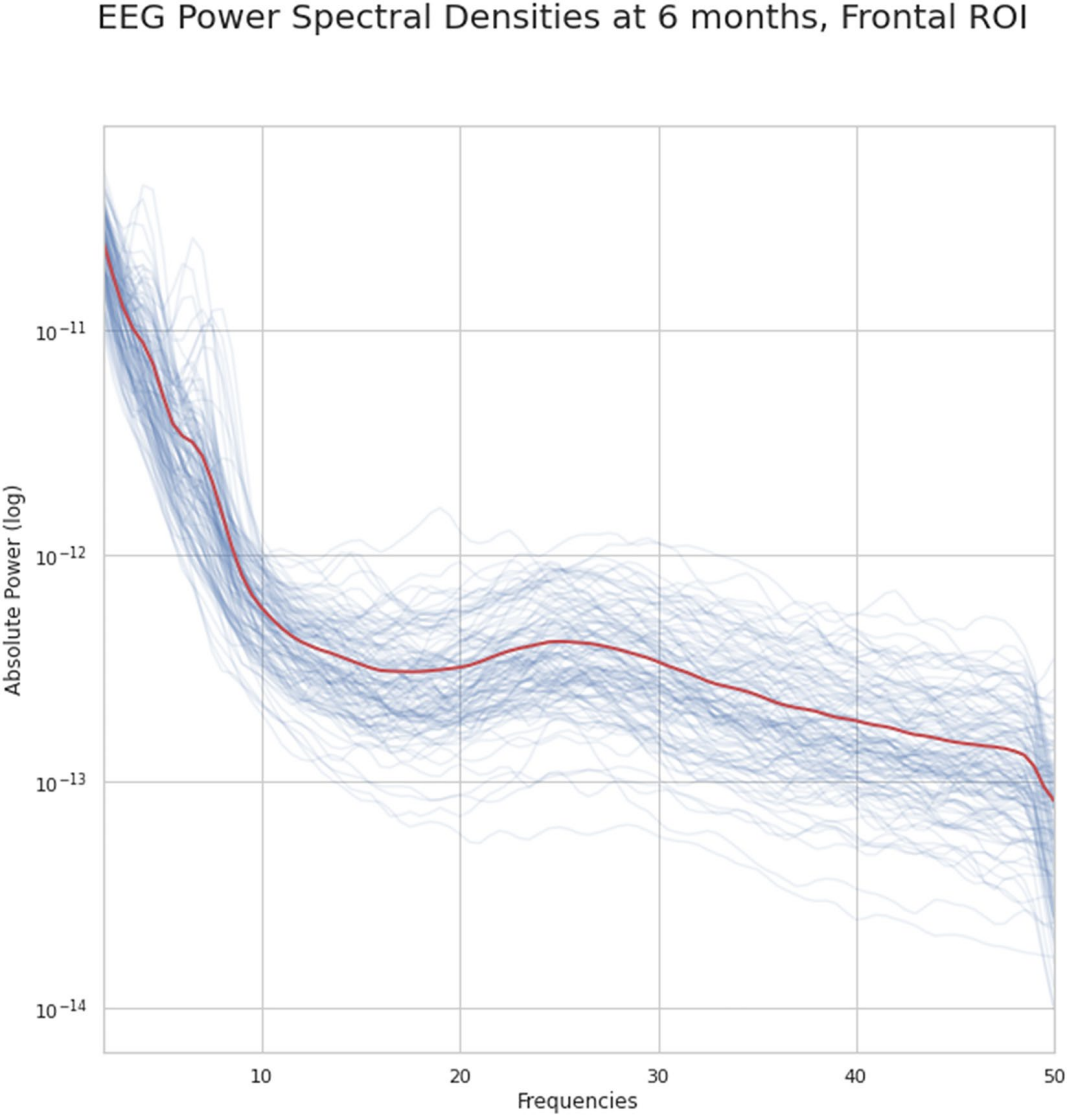
Absolute power spectral densities at 6 months for each EEG recording. Each thin blue line represents spectral power density (averaged across the 3 frontal channels) of an individual recording, while the thick red line represents the average across recordings.

For our analysis, we interpolated the EEG to the international 10–20 montage, and selected three frontal channels: F3, Fz, and F4. Spectral power in the frontal region differs between infant‐siblings and control infants, and appears to be functionally related language and cognition (Benasich et al., 2008; Fox & Bell, 1990; Hodge et al., 2010; Huberty et al., 2021; Jones et al., 2020; Levin et al., 2017). The frequency bands selected for analysis included delta (2–4 Hz), theta (4–6 Hz), low‐alpha (6–9 Hz), high‐alpha (9–13 Hz), beta (13–30 Hz), and gamma (30–50 Hz). For each electrode in the region of interest (ROI), a power spectral density (PSD) was computed with the Welch method using the *pwelch* function in MATLAB with 4s epochs (0.25 Hz frequency bin resolution) and a Hanning window with 50% overlap to account for the fact that the Hanning window weighs the center of the data segment more heavily than the sidelobes. The total power was computed for each frequency band using the trapezoidal method. The PSDs averaged across all three frontal electrodes for participants' recordings can be viewed in Figure 1.

### 2.3 Statistical analysis

All analyses were performed in Python 3.8.10 using Jupyterlab notebooks hosted on a JupyterHub service provided by the Calcul Quebec (https://www.calculquebec.ca) and Compute Canada (www.computecanada.ca) national high‐performance computing infrastructure. Data manipulation was done using the Pandas 1.2.3 Python library and statistical analyses were conducted by interfacing STATA 17 through Python using PyStata.

**FIGURE 2:**
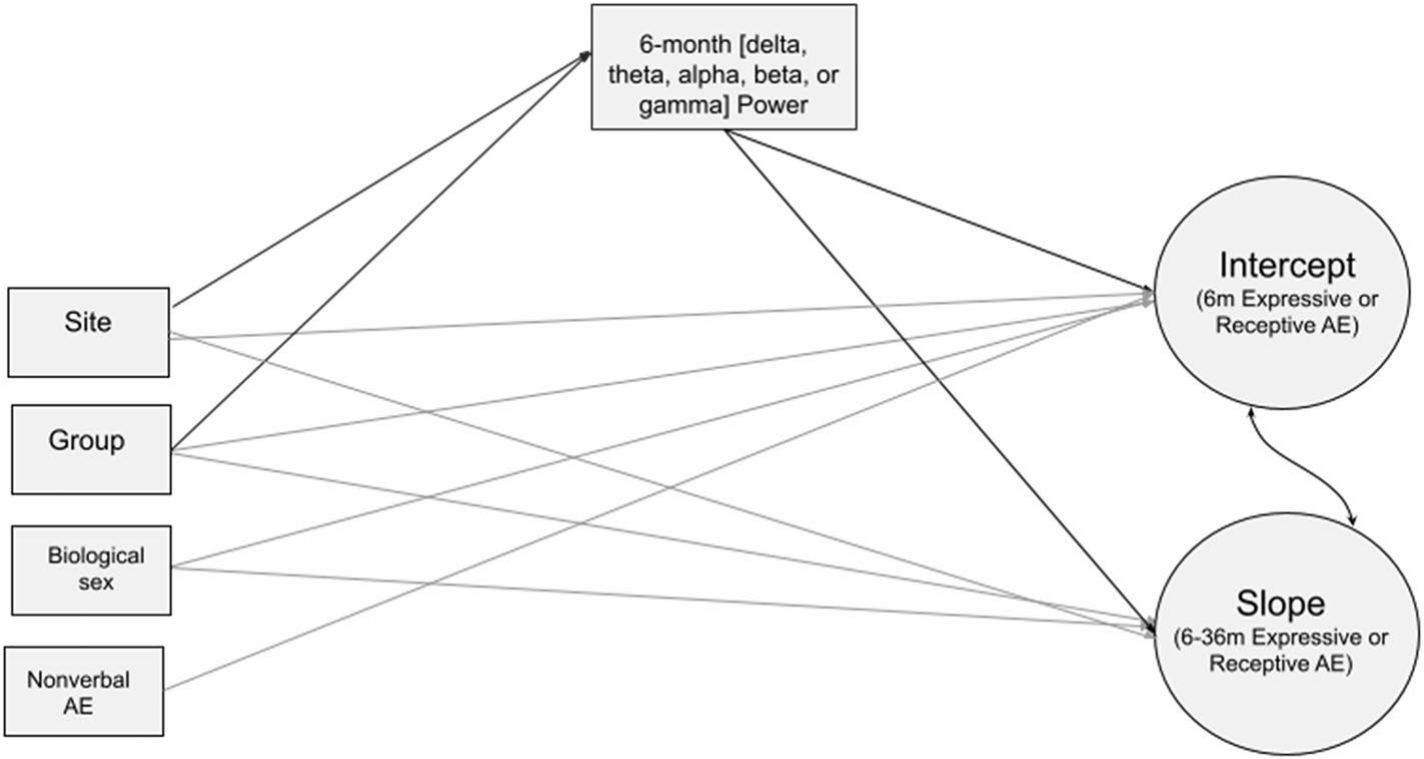
Schematic representation of the growth curve model to test concurrent and longitudinal associations between EEG power and receptive and expressive language development. In this schema, circles indicate the latent outcome variables, and squares indicate observed predictor variables. Arrows indicate paths between variables. The curved line between intercept and slope indicates that they were allowed to covary.

Two growth curve models (GCM) were fit to the measurements of MSEL Expressive and Receptive Language AE scores respectively, specifying latent factors for the intercept (Receptive/Expressive AE at 6 months) and slope (the change in Receptive/Expressive AE between 6 and 36 months). Modeling the intercept and slope as latent variables provides important advantages; average slopes are not assumed to be linear, and latent variables can minimize measurement error (Curran et al., 2010). To aid convergence of our final model, estimates of individual scores on intercept and slope were extracted and entered into our specified model (Figure 2). The model tested whether absolute power was significantly associated with intercept and slope, while controlling for effects of site, group (coded 0 = control infant; 1 = infant‐sibling), sex (coded 0 = Male; 1 = Female) and 6‐month nonverbal age equivalent score, since concurrent nonverbal and verbal ability are thought to be associated (Messinger et al., 2013). A separate model was run for each frequency band and MSEL language scale, for a total of 12 models. For models with a significant association between EEG frequency band power and language, secondary models were run with an interaction term between group and EEG power on intercept and slope.

## 3 RESULTS

### 3.1 Sample characteristics

**FIGURE 3:**
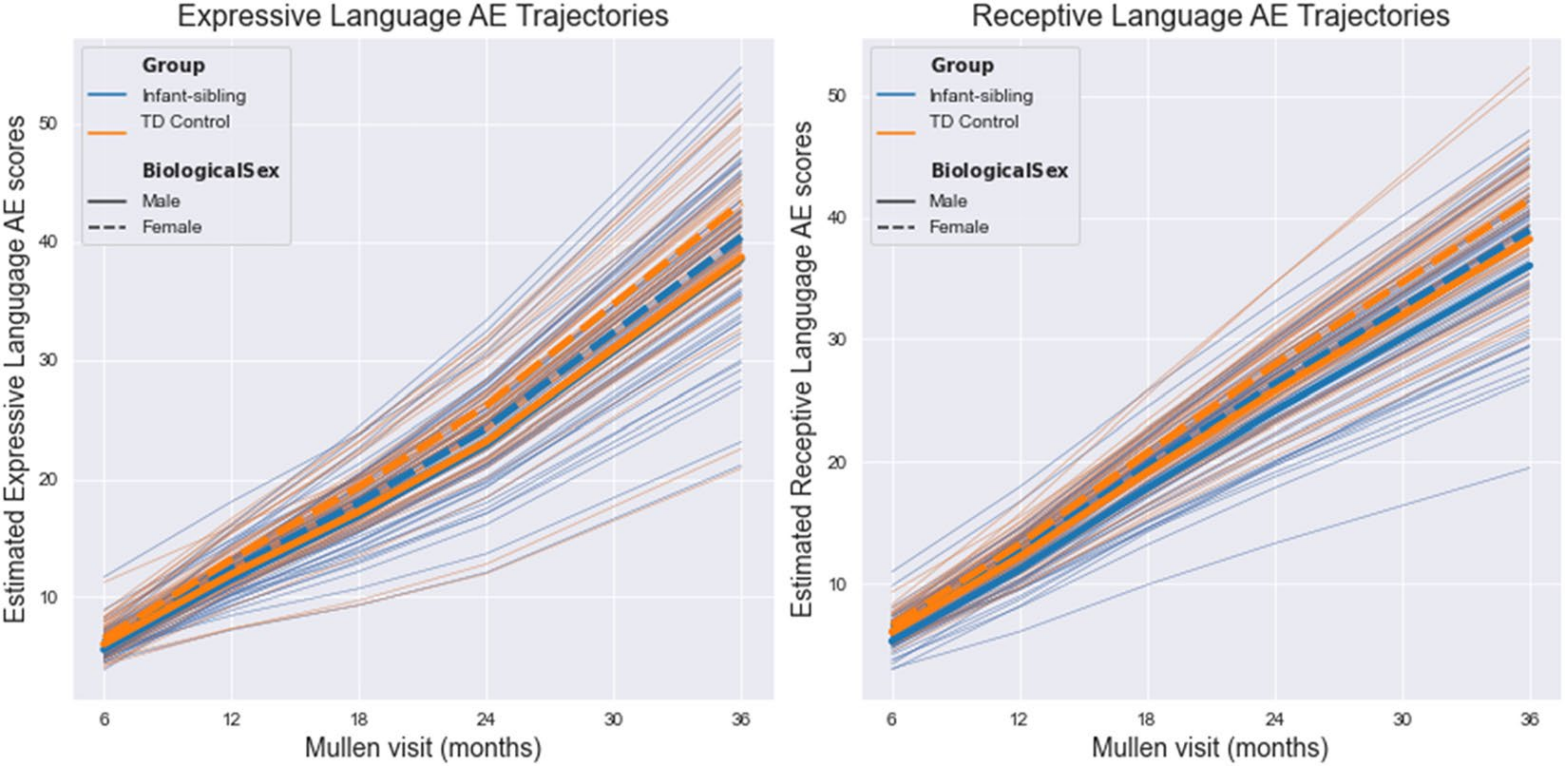
Expressive and Receptive Language AE by biological sex and groupacross all visits. Group average trajectories are represented by the thick bolded lines, while the thin lines represent individual fitted trajectories.

We report in Table 1 the sample means and standard deviations for age and MSEL Receptive and Expressive Language AE scores at each visit. Age at MSEL assessment did not differ between the infant‐sibling and control groups, while the infant‐sibling group had lower Expressive and Receptive Language AE scores. Trajectories for Receptive and Expressive Language, split by biological sex and group status, can be viewed in Figure 3. Regression coefficients, standard errors, z‐scores, *p*‐values, and confidence intervals for each growth curve model can be viewed in Tables 2 and 3. Distributions of 6‐month EEG power, and MSEL Receptive and Expressive Language AE at each visit can be viewed in Supplemental Figures S1 and S2 in the appendices. Fit indices for models can be viewed in the supplementary materials Table S2.

**TABLE 2:** Growth Curve Model results for Expressive Language AE. Both unstandardized and standardized coefficients are reported, where standardized coefficients can be used to compare the effect size between tests.

	Coefficient unstandardized/standardized		Std error	Z‐Score	*p* value	95% conf. interval
Intercept of expressive language						
Delta	0.501	0.075	0.424	1.182	0.237	[−0.33, 1.331]
Theta	0.312	0.063	0.345	0.905	0.366	[−0.364, 0.988]
Low‐alpha	0.558	0.126	0.306	1.823	0.068	[−0.042,1.157]
High‐alpha	**1.119**	**0.159**	**0.475**	**2.357**	**0.018**	[**0.189, 2.049**]
Beta	0.745	0.121	0.413	1.805	0.071	[−0.064, 1.554]
Gamma	0.402	0.074	0.369	1.090	0.276	[−0.321, 1.124]
Group	−0.248	−0.096	0.165	−1.502	0.133	[−0.572, 0.076]
Site	0.249	0.096	0.197	1.268	0.205	[−0.136, 0.635]
biological sex	−0.066	−0.025	0.171	−0.385	0.701	[−0.4, 0.269]
6‐month nonverbal AE	**0.426**	**0.626**	**0.051**	**8.279**	**<0.001**	[**0.324, 0.526**]
Slope of expressive language development						
Delta	−0.259	−0.042	0.526	−0.492	0.623	[−1.289, 0.772]
Theta	0.076	0.016	0.397	0.191	0.848	[−0.702, 0.854]
Low‐alpha	0.095	0.023	0.371	0.258	0.797	[−0.630, 0.821]
High‐alpha	0.280	0.042	0.588	0.477	0.633	[−0.871.1.432]
Beta	0.062	0.010	0.508	0.122	0.903	[−0.934, 1.058]
Gamma	−0.032	−0.001	0.438	−0.070	0.942	[−0.891, 0.827]
Group	−0.207	−0.087	0.203	−1.016	0.309	[−0.606, 0.192]
Biological sex	**0.559**	**0.235**	**0.203**	**2.754**	**0.006**	[**0.161, 0.957**]

*Note*: Biological sex was coded as (0 = Male, 1 = Female), group was coded as (0 = control infant, 1 = infant‐sibling).

**TABLE 3:** Growth curve model results for receptive language AE. Both unstandardized and standardized coefficients are reported, where standardized coefficients can be used to compare the effect size between tests.

	Coefficient unstandardized/standardized		Std error	*Z*‐Score	*p* value	95% conf. interval
Intercept of receptive language						
Delta	0.473	0.089	0.368	1.286	0.199	[−0.248, 1.194]
Theta	0.362	0.091	0.295	1.229	0.219	[–0.215, 0.939]
Low‐alpha	0.145	041	0.266	0.544	0.586	[−0.377, 0.667]
High‐alpha	0.659	0.117	0.415	1.587	0.113	[−0.155, 1.472]
Beta	0.187	0.038	0.363	0.515	0.606	[−0.525, 0.899]
Gamma	0.220	051	0.320	0.690	0.492	[−0.407, 0.846]
Group	**−0.287**	**−0.140**	**0.144**	**−1.998**	**0.046**	[**−0.569, −0.005**]
Site	**−0.335**	**−0.161**	**0.154**	**−2.182**	**0.029**	[**−0.636, −0.034**]
Biological sex	**0.469**	**0.229**	**0.147**	**3.182**	**0.001**	[**0.18, 0.757**]
6‐month nonverbal AE	**0.295**	**0.546**	**0.040**	**7.361**	**<0.001**	[**0.217, 0.374**]
Slope of receptive language development						
Delta	0.069	014	0.390	0.177	0.859	[−0.697, 0.835]
Theta	0.119	0.034	0.293	0.407	0.683	[−0.455, 0.694]
Low‐alpha	0.194	0.062	0.273	0.713	0.475	[−0.341, 0.731]
High‐alpha	0.477	0.096	0.433	1.102	0.270	[−0.372, 1.328]
Beta	−0.025	−0.005	0.378	−0.066	0.946	[−0.768, 0.717]
Gamma	−0.051	−0.013	0.326	−0.160	0.875	[−0.691, 0.589]
Group	**−0.363**	**−0.201**	**0.151**	**−2.405**	**0.016**	[**−0.66, −0.067**]
Biological sex	**0.487**	**0.270**	**0.150**	**3.232**	**0.001**	[**0.192, 0.783**]

### 3.2 Predictors of expressive language AE intercept

Six‐month Nonverbal AE and 6‐month high‐alpha EEG power predicted Expressive Language AE intercept (6‐month visit Expressive Language AE). Higher alpha power (*b* = 1.119, 95% CIs = [0.189–2.049], *p* = 0.018) and higher nonverbal AE (*b* = 0.426, 95% CIs = 0.324–0.526, *p* < 0.001) were associated with higher Expressive Language at the 6‐month visit (see Table 2). To ensure that variation in the chronological age at the 6‐month assessment did not account for the association between alpha power and intercept, a supplementary model was run, with an additional term between chronological age at assessment and intercept (supplementary Table S3).

### 3.3 Predictors of expressive language AE slope

Biological sex was a significant predictor of the slope of Expressive Language (*b* = 0.559, 95% CIs = [0.161,0.957], *p* = 0.006), with being female associated with a steeper slope. Neither group (*b* = −0.207, 95% CIs = [–0.606,0.192], *p* = 0.309), nor absolute power in any of the defined frequency bands (all *p* values > 0.05) were significant predictors of Expressive Language slope.

### 3.4 Predictors of receptive language AE intercept

Six‐month nonverbal AE, biological sex, and group predicted Receptive Language AE intercept. Being female (*b* = 0.469, CIs = [0.18, 0.757], *p* = 0.001), being in the control group (*b* = −0.287, CIs = [−0.569, –0.005], *p* = 0.046), and having higher 6‐month nonverbal AE (*b* = 0.295, CIs = [0.217, 0.374], *p* < 0.001) were each associated with higher Receptive Language AE at the 6‐month visit (see Table 3). None of the frequency bands were associated with Receptive Language intercept.

### 3.5 Predictors of receptive language AE slope

Being female (*b* = 0.488, CI's = [0.192, 0.783], *p* = 0.001) and being in the control group (*b* = −0.364, CI's = [−0.66, −0.067], *p* = 0.016) were significantly associated with steeper slopes of Receptive Language. None of the frequency bands were associated with Receptive Language slope (all *p* values > 0.05, Table 3).

### 3.6 Interaction between group (infant‐sibling/control) s and absolute power on intercept and slope

To test whether the association between high‐alpha power and concurrent Expressive Language differed between infant‐siblings and controls, we ran additional supplemental models which included a group‐by‐high‐alpha EEG power interaction term as a predictor of Expressive Language intercept and slope. The interaction terms between group and high‐alpha power was not statistically significant (*b =* −0.104, *p* = 0.909, 95% CI = [−1.888, 1.681]), suggesting that the association does not differ between the infant‐sibling and control groups.

## 4 DISCUSSION

This study tested whether spectral EEG power within the first year of life is associated with concurrent language ability or its developmental change between 6 and 36 months in infant‐siblings compared to typically developing control infants without first‐degree familial history of autism. We specifically focused on possible neural mechanisms underlying heterogenous language development in autism. Additionally, our model controlled for nonverbal cognitive ability to ensure that any significant associations were specific to language. Neural oscillations at distinct frequencies are increasingly being related to a number of basic and higher cognitive faculties (Murphy, 2015), and our results are in line with previous findings that reported an association between EEG power in the alpha band and expressive but not receptive language ability (Levin et al., 2017), with higher spectral power at 6 months being associated with higher concurrent language ability. Some prior studies have reported associations between gamma power and language (Benasich et al., 2008; Wilkinson et al., 2019). While our model did not report an association between gamma and language scores, differences in sample ascertainment and study design could account for some differences. For example, The sample in the study by Benasich et al. (2008) were infants at risk for specific language impairment, and the study by Wilkinson et al. (2019) was interested in whether change in gamma power trajectories predicted language at a specified outcome timepoint.

Oscillatory activity in the alpha range has been suggested to be associated with both speech generation (Wojtecki et al., 2017) and language comprehension (Wöstmann et al., 2017), possibly by facilitating the integration of multi‐modal information streams, and the inhibition of distractor stimuli (van Driel et al., 2014). During periods of sustained attention, alpha‐band desynchronization (resulting in reduced spectral power) and synchronization (increased spectral power) may occur in different brain regions depending on whether the region is functionally relevant to the modality of the stimuli (Orekhova et al., 2001; Wöstmann et al., 2017). We did not, however, find an association between alpha power and receptive language. It may be that greater variability in expressive language scores than receptive language scores at 6 months made it relatively easier to identify associations with expressive language. Alternatively, receptive language has been suggested to rely on complex networks of brain activity (Gaudet et al., 2020), and EEG spectral power may be too broad a measure to map on to the development of this domain.

While alpha power's association to language may be confounded by its association to attention (given that attentional processes are associated with cognitive performance; Breznitz & Friedman, 1988; Klimesch, 2012), we note that we included nonverbal AE as a covariate in our model, suggesting the association between alpha power and expressive language was relatively specific, rather than being due to variation in general cognitive ability. Still, it may be that alpha power is functionally important to language precisely because of its role in facilitating attentional control during speech. This is in contrast to other metrics of alpha oscillatory activity, such as peak alpha frequency, which appears to be a stronger indicator of non‐verbal cognition, but not language (Carter Leno et al., 2021; Dickinson et al., 2018). Studies that test associations between different metrics of alpha oscillations (resting power, evoked desynchronization, peak frequency), and attentional control and language ability would help to better understand the functional role of alpha oscillations in language comprehension and expression.

Our findings also suggest some continuity in the neural processes of language in typical and atypical development. While the infant‐sibling group had significantly lower language ability than the control group at various points in development between 6 and 36 months, the association between EEG spectral power and language ability was not moderated by group (although we note that testing interaction terms requires more statistical power). Still, this was as we hypothesized and as observed in a broad range of populations, from typically developing infants (Brito et al., 2016; Cantiani et al., 2019; Levin et al., 2017; Pierce et al., 2021; Saby & Marshall, 2012), to those at increased likelihood for various neurodevelopmental and genetic conditions (Benasich et al., 2008; Gou et al., 2011; Jones et al., 2020; Levin et al., 2017; Wilkinson et al., 2019; Wilkinson et al., 2019). Despite the quantitative differences in spectral power that have been observed between infant‐siblings and control infants, its association to language appears to be similar between the two groups.

By modeling rates of language development using data from infants followed longitudinally over multiple visits, the current study expands upon previous studies that reported associations between EEG power and language assessed at single points in development. While we found that EEG alpha power was associated with concurrent expressive language ability, we did not find evidence of longitudinal associations between alpha power and the development of language. Our findings suggest that alpha power may be a correlate of language ability but not a driver of its developmental course. One possible explanation for this is that other environmental and biological factors may influence language development alongside individual variation in alpha power. The association between alpha power and language may be weakened as infants who appear to have lower power at 6‐months nonetheless gain language skills throughout development.

Our study has important limitations to consider. First, we controlled for MSEL nonverbal scores in our models, however in infancy it is more difficult to delineate general cognitive ability and language, and in our model nonverbal DQ was highly associated with both expressive and receptive language. Thus, including the nonverbal DQ covariate may have made it more difficult to detect associations between EEG and language. Second, although SEM reflects some model specifications and thus encourages grounding in theoretical frameworks on a priori hypotheses, we acknowledge our analyses include multiple uncorrected statistical tests. We note that if we were to adjust our reported *p*‐values using a false discovery rate correction, the association between high‐alpha power and intercept would no longer reach significance (*p* = 216). Therefore, it is important that these findings are replicated before any stronger conclusions are drawn. Finally, our study was limited in the ability to assess some other factors known to influence early development, such as the degree of genetic risk for autism (other than family history) and socio‐economic status, and future studies could assess whether these factors influence the development of language alongside EEG metrics of cortical activity (D’Abate et al., 2019; McDonald et al., 2019; Noble et al., 2007). Other measures of oscillatory activity that capture temporal (cross‐frequency coupling) and spatial (inter‐regional connectivity properties of spectral power) have also been associated with language (Hermes et al., 2014; Lizarazu et al., 2019), but haven't been applied in early infancy. These measures reflect aspects of cortical development, such as cortico‐cortical connectivity, that are affected in individuals with autism (O’Reilly et al., 2017), and may be stronger indicators of later behavioral development.

## CONFLICT OF INTEREST STATEMENT

Authors have no financial conflicts of interest to declare.

## Supporting information

Supporting Information S1

## Data Availability

Due to restrictions involving institutional data‐sharing agreements between McGill University and each dataset's institution of origin, data that support the findings of this study is not publicly available. The code used for the analyses is available upon reasonable request to the corresponding author.

## Footnotes

^1^
We note that this biological sex ratio differs from previously estimated ratios for infant‐siblings (3.18 odds ratio, male to female; Messinger et al., 2013).
