# Supplementary material for: Neural mechanisms of language development in infancy
Source: Infancy. 2023 Mar 21;28(4):754–70. doi: 10.1111/infa.12540 (PMC10947526; doi:10.1111/infa.12540)
Supplement: Supplementary file 1 — Supporting Information S1 [file INFA-28-754-s001.docx]

## Appendices

Supplementary Table 1: Breakdown of the count of MSEL assessments by site and group (top), and the number of participants who received at least 1, 2, 3, and 4 longitudinal mullen assessments.

| **Site** | **Group** | **MSEL 6m** | **MSEL 12m** | **MSEL 18m** | **MSEL 24m** | **MSEL 36m** |
| --- | --- | --- | --- | --- | --- | --- |
| **London** | Increased Likelihood | 29 | 29 | 0 | 29 | 29 |
|  | TD control | 26 | 26 | 0 | 26 | 26 |
| **Seattle** | Increased Likelihood | 34 | 34 | 33 | 32 | 0 |
|  | TD control | 42 | 38 | 37 | 4 | 0 |

| Number of repeated  MSEL assessments | **N** |
| --- | --- |
| 4 | 90 |
| 3 | 35 |
| 2 | 3 |
| 1 | 3 |

|  |
| --- |
| 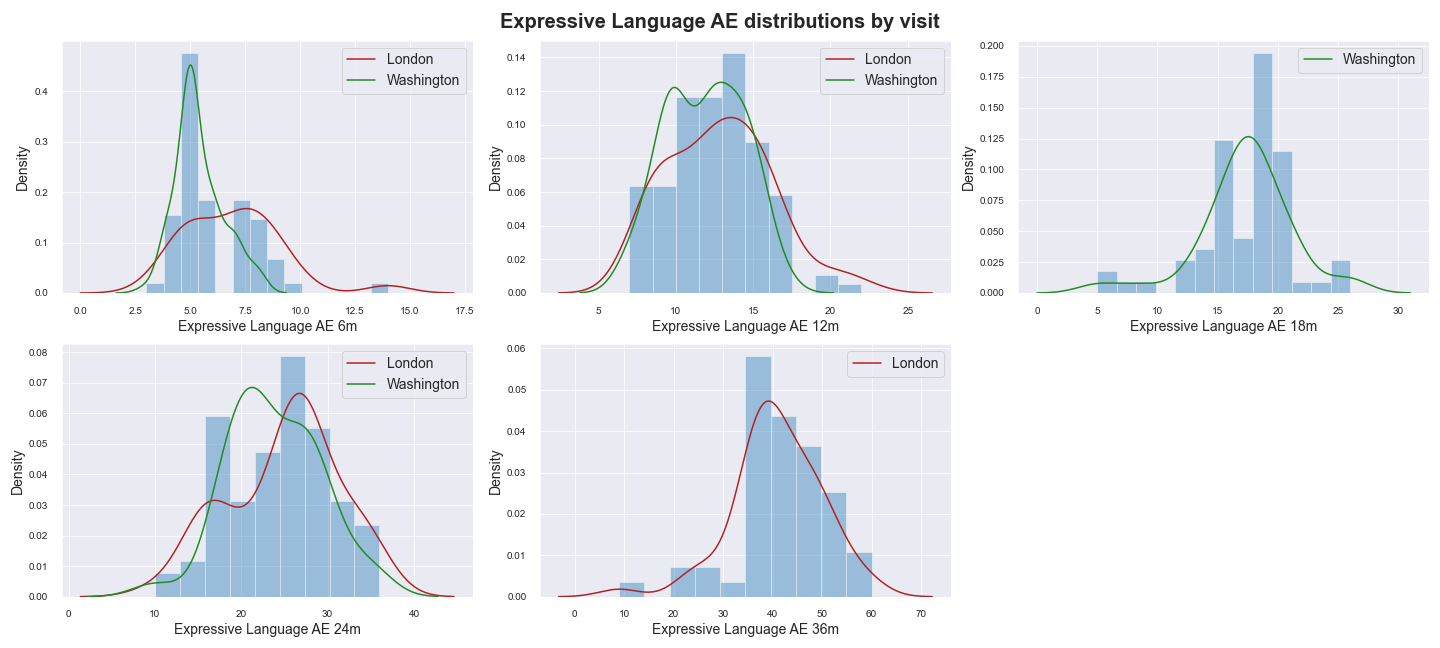  Supplemental Figure 1. Histograms of the distributions of Expressive Language AE (top) and Receptive Language AE (bottom) at each visit. Blue vertical bars represent the entire sample, while the red and green lines represent the distribution for each site. |

####

####

####
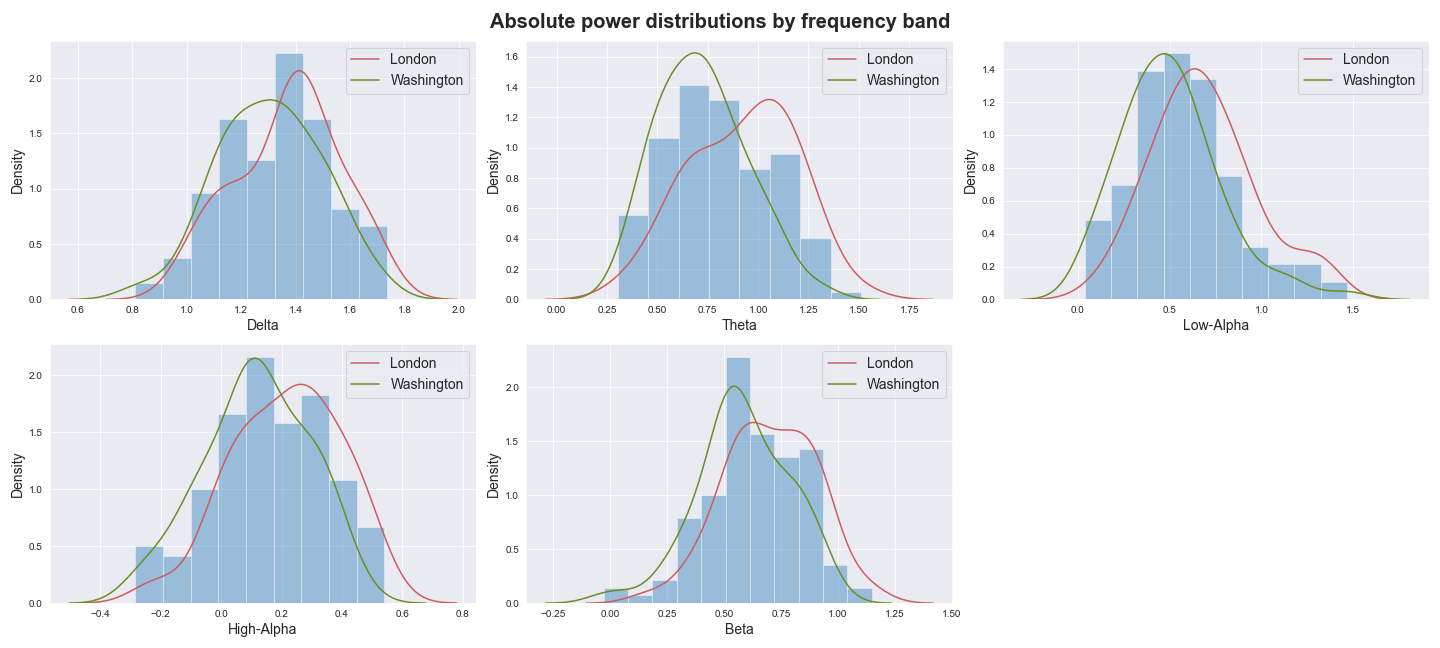

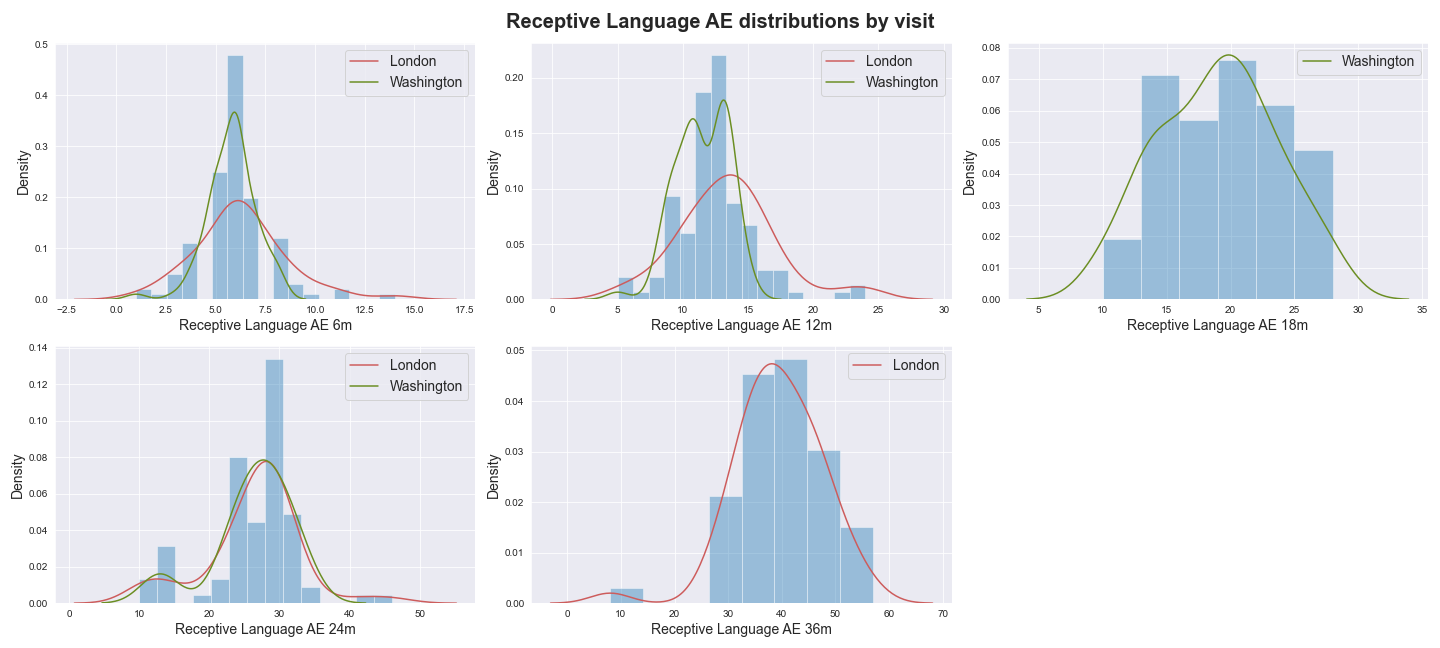


#### Supplemental Figure 2. Histograms of the distributions of 6-month absolute power in each frequency band. Blue vertical bars represent the entire sample, while the red and green lines represent the distribution for each site.

Supplementary table 2: fit indices for models: for the TLI, values closer to 1 indicate a better fit. For the RMSEA, upper band values at .1 or below generally indicate good fit.

| Expressive Language | | |
| --- | --- | --- |
|  | RMSEA [lower bound, upper bound[ | TLI |
| Delta | 0, [0, 0.048] | 1.119 |
| Theta | 0; [0, 0.063] | 1.091 |
| Alpha | 0.1; [0.018, .177] | 0.82 |
| Alpha | 0.086; [0, .165] | 0.864 |
| Beta | 0.074; [0, .156] | 0.895 |
| Gamma | 0; [0, 0.121] | 1.002 |
| Receptive Language | | |
| Delta | 0.023; [0, .126] | 0.991 |
| Theta | 0.025; [0, .126] | 0.991 |
| Low Alpha | 0.123; [0.053, 0.197] | 0.776 |
| High Alpha | 0.109;  [0.035, 0.185] | 0.817 |
| Beta | 0.108; [0.033, 0.185] | 0.818 |
| Gamma | 0.081; [0, 0.162] | 0.901 |

Supplementary table 3: Growth Curve Model results for Expressive Language AE and High Alpha power, with an additional term included between chronological age at assessment and intercept.

|  | Coefficient | Std error | Z-Score | p value | 95% conf. interval |
| --- | --- | --- | --- | --- | --- |
| Intercept on |  |  |  |  |  |
| High Alpha | 0.97 | 0.409 | 2.37 | 0.018 | [0.167, 1.772] |
| Site | 0.5669109 | 0.1678169 | 3.38 | 0.001 | [0.238, 0.896] |
| Biological sex | -0.1816252 | 0.1481321 | -1.23 | 0.22 | [-0.472, 0.109] |
| Group | 0.0236361 | 0.1397245 | 0.17 | 0.866 | [-0.25, 0.297] |
| 6-month Nonverbal AE | 0.483852 | 0.0433616 | 11.16 | < . 001 | [0.399, 0.569] |
| Slope on |  |  |  |  |  |
| High Alpha | 0.2316982 | 0.5914111 | 0.39 | 0.695 | [-0.927, 1.391] |
| biological sex | 0.4818275 | 0.2129973 | 2.26 | 0.024 | [0.064, 0.899] |
| Group | -0.3454591 | 0.2039303 | -1.69 | 0.09 | [-0.745, 0.054] |
